# The impact of binaural beats on creativity

**DOI:** 10.3389/fnhum.2013.00786

**Published:** 2013-11-14

**Authors:** Susan A. Reedijk, Anne Bolders, Bernhard Hommel

**Affiliations:** Institute for Psychological Research and Leiden Institute for Brain and Cognition, Leiden UniversityLeiden, Netherlands

**Keywords:** creativity, binaural beats, gamma, alpha, cognitive enhancement

## Abstract

Human creativity relies on a multitude of cognitive processes, some of which are influenced by the neurotransmitter dopamine. This suggests that creativity could be enhanced by interventions that either modulate the production or transmission of dopamine directly, or affect dopamine-driven processes. In the current study we hypothesized that creativity can be influenced by means of binaural beats, an auditory illusion that is considered a form of cognitive entrainment that operates through stimulating neuronal phase locking. We aimed to investigate whether binaural beats affect creative performance at all, whether they affect divergent thinking, convergent thinking, or both, and whether possible effects may be mediated by the individual striatal dopamine level. Binaural beats were presented at alpha and gamma frequency. Participants completed a divergent and a convergent thinking task to assess two important functions of creativity, and filled out the Positive And Negative Affect Scale—mood State questionnaire (PANAS-S) and an affect grid to measure current mood. Dopamine levels in the striatum were estimated using spontaneous eye blink rates (EBRs). Results showed that binaural beats, regardless of the presented frequency, can affect divergent but not convergent thinking. Individuals with low EBRs mostly benefitted from alpha binaural beat stimulation, while individuals with high EBRs were unaffected or even impaired by both alpha and gamma binaural beats. This suggests that binaural beats, and possibly other forms of cognitive entrainment, are not suited for a one-size-fits-all approach, and that individual cognitive-control systems need to be taken into account when studying cognitive enhancement methods.

## Introduction

Creativity is an important skill in the human cognitive repertoire, it is useful in art and science and essential in day-to-day life. Unfortunately, however, research into creativity is rather cluttered and mechanistic models about how creativity might work are not available (Dietrich and Kanso, 2010). It is thus not surprising that there is no single, widely accepted definition of creativity. What can be said, though, is that many cognitive processes seem to be involved, and that sub-functions underlying creativity depend on both state (Baas et al., 2008; Davis, 2009) and trait (Akbari Chermahini and Hommel, 2010) characteristics. Of all the processes involved in creativity, Guilford (1950, 1967) identifies divergent and convergent thinking as its two main ingredients. Together with insight (a possible sub-component of convergent thinking; see Bowden et al., 2005), these are nowadays still considered the most important processes in creativity (Dietrich and Kanso, 2010). Accordingly, it was these two processes that we considered in the present study.

Both divergent and convergent thinking have been assumed to be influenced by positive mood (e.g., Baas et al., 2008; Davis, 2009), but the mechanism underlying this impact remains unclear. Based on the observation that schizophrenic patients, who suffer from an overdose of the neurotransmitter dopamine (for a review, see Davis et al., 1991), sometimes exhibit extraordinary creative performances (Keefe and Magaro, 1980; Nelson and Rawlings, 2008), some authors have assumed a strong link between creativity and dopamine (Eysenck, 1993). Indeed, positive-going mood is accompanied by phasic changes in the production and availability of dopamine in the mesolimbic and nigrostriatal systems of the brain (Ashby et al., 1999), which again is likely to facilitate cognitive search operations and related processes underlying creative behavior (Akbari Chermahini and Hommel, 2010; Hommel, 2012). If so, factors or techniques that are likely to modulate dopamine production or transmission could be suspected to have an impact on cognitive operations underlying creativity.

One phenomenon that has been suspected to propagate creativity is known under the name of “binaural beats”, an auditory illusion that can be considered a kind of cognitive or neural entrainment (Vernon, 2009; Turow and Lane, 2011). This phenomenon has encouraged sweeping claims about mind enhancement, and some websites even went as far as calling the illusion a “digital drug”. While binaural beats indeed seem to exert some effect on cognitive functioning and mood (Lane et al., 1998), and on neural firing patterns in the brain (Kuwada et al., 1979; Karino et al., 2006; Pratt et al., 2009; but see Vernon et al., 2012), it is as yet unclear how they do so. The binaural-beat illusion arises when two tones of a slightly different frequency are each presented to different ears. For instance, when a tone of 335 Hz is presented to the right ear and a tone of 345 Hz to the left ear, this results in a subjectively perceived binaural beat of 10 Hz. Hence, instead of hearing two different tones, most individuals will hear just one tone that fluctuates in frequency or loudness: a beat (Oster, 1973).

How exactly the brain produces the perception of these beats is unclear, but the reticular activation system and the inferior colliculus seem to play a role (Kuwada et al., 1979; McAlpine et al., 1996; Turow and Lane, 2011). In animals, binaural-beat producing stimulus conditions have been shown to produce particular neural patterns of phase locking, or synchronization, beginning in the auditory system and propagating to the inferior colliculus (Kuwada et al., 1979; McAlpine et al., 1996). Even though the neural response to objectively presented beats is stronger, binaural beats seem to elicit similar neural responses in both humans and animals (Kuwada et al., 1979; McAlpine et al., 1996; Schwarz and Taylor, 2005; Karino et al., 2006), suggesting that the illusion arises through pathways normally associated with binaural sound detection (Kuwada et al., 1979; Pratt et al., 2010). As in humans binaural beats have been found to affect cognitive functioning and mood (Lane et al., 1998; Vernon, 2009), and responses to binaural beats are detectable in the human EEG (Schwarz and Taylor, 2005; Pratt et al., 2009), it can be assumed that neuronal phase locking spreads from the auditory system and the inferior colliculus over the cortex. A spreading pattern of neuronal activation and synchronization might affect short- and long-distance communication in the brain, processes which depend on neuronal synchronization and, presumably, on particular neurotransmitter systems (Schnitzler and Gross, 2005), thus affecting cognitive processing.

If binaural beats affect cognition through neural synchronization, it is possible that the frequency of the beat matters. For instance, short-range communication within brain areas is often associated with neural synchronization in the gamma frequency, while long-range communication is associated with neuronal phase locking in the slower frequency bands (von Stein and Sarnthein, 2000; Schnitzler and Gross, 2005). Moreover, a variety of frequency bands have been considered to represent the “messenger frequency” of cognitive-control signals. For instance, synchronization in the gamma frequency range seems to play a role in the top-down control of memory retrieval (Keizer et al., 2009), which should be relevant for many creativity tasks. Also of interest, phase locking in the alpha band has been associated with lower cortical arousal in general (Fink and Neubauer, 2006) and enhanced top-down control in creativity-related performance in particular (von Stein and Sarnthein, 2000; Fink et al., 2009). Especially divergent thinking seems to be associated with alpha wave synchronization (Fink et al., 2006, 2009). It could therefore be reasoned that inducing a state of lower cortical arousal by presenting people with alpha frequency binaural beats temporarily increases their performance on a divergent thinking task. Given that the available evidence highlights the alpha and gamma bands as possible messenger frequencies of control signals in creativity-related tasks, we investigated whether binaural beats presented at these two frequencies might affect performance in convergent- and divergent-thinking tasks—as compared to a control condition.

Performance in creativity tasks does not only depend on current states but is also affected by trait variables. As suggested by Eysenck (1993) and Ashby et al. (1999), creative performance seems to depend on an individual’s basic supply of (striatal) dopamine. This suggestion fits with recent ideas about the interaction of frontal and striatal dopaminergic pathways in generating cognitive control. According to Cools and d’Esposito (2009), the frontal dopaminergic pathway (originating in the Ventral Tegmental Area) supports focusing on the current task while the striatal pathway (originating in the Substantia Nigra) facilitates the mental flexibility and switching between mental representations. Considering that this latter ability is particularly relevant for divergent thinking, it is not surprising that divergent thinking, but not convergent thinking, was found to be related to the spontaneous eye-blink rate (EBR; Akbari Chermahini and Hommel, 2010)—a clinical marker of striatal dopaminergic functioning (Karson, 1983; Shukla, 1985; Taylor et al., 1999).

Importantly for our study, markers of the individual striatal dopamine level (EBR) do not only predict individual performance in a divergent-thinking task, but also whether and how individuals are affected by state variables. Only recently, Akbari Chermahini and Hommel (2012) demonstrated that the creativity-enhancing effect of positive mood was restricted to individuals with low EBRs, i.e., low striatal dopamine levels. Indeed, tonic and phasic effects of neurotransmitters have often been assumed to interact in nonlinear fashions, in such a way that phasic changes can be more easily detected or are otherwise more effective if combined with a relatively low tonic baseline (e.g., Grace, 1991; Cohen et al., 2002). If so, and assuming EBRs reflect a fairly stable baseline level of tonic and phasic dopamine activity in the striatum, the hypothetical creativity-enhancing impact of binaural beats would be expected to be visible mainly in individuals with relatively low EBRs. We tested this hypothesis by analyzing performance in convergent- and divergent-thinking tasks, and beat-induced changes therein, as a function of low versus high EBR.

## Methods

Twenty-four first-year psychology or educational studies students (22 female, 2 male; 17–25 years) of Leiden University participated in exchange for course credit and/or pay. All participants had normal or corrected-to-normal sight and normal hearing, and no history of epileptic attacks or other neuropsychological illnesses. All participants were tested between 1 pm and 7 pm, and for each participant all sessions took place at the same time of the day. This was done to reduce variation due to normal daily fluctuations in EBR, mood, and related variables.

After the study procedure was explained to them by the experimenter, written informed consent was obtained from all participants. In the case of one underage participant, written informed consent was also obtained from the parents/caretakers. Participants were not made aware of the goal of the study beforehand, but all were debriefed after completing all sessions. The study was approved by the Leiden University Ethics Committee of the Institute of Psychology.

**Figure 1:**
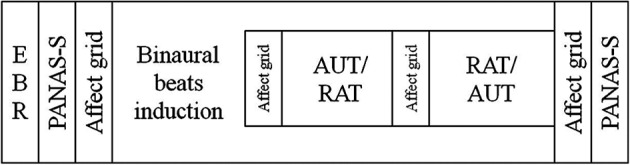
**Diagram of the task order in every session.** Participants always completed the tasks in this order, regardless of beat frequency. Whether a participant completed the alternate uses task (AUT) before the remote associations task (RAT) (or vice versa) was randomized across participants.

Participants came in for three sessions: one in which they were exposed to alpha frequency (10 Hz) binaural beat stimuli (the Alpha condition), one in which they were exposed to gamma frequency (40 Hz) binaural beat stimuli (the Gamma condition), and one in which they listened to a constant tone of 340 Hz (the Control condition). The order of these three conditions and the two creativity tasks was counterbalanced across participants by means of a Latin square design. In every session a participant would complete the same tasks in the same order but with different items (see below) to avoid learning effects. The order of the items within each session was the same for all participants. Before starting the tasks, spontaneous EBRs were measured, and participants listened to a 3 min sound file (inducing the binaural beats, or the control sound) during which they did not complete any task. While the sound file continued to play, participants then carried out the creativity tasks using pencil and paper. At the beginning and end of the session participants’ positive (PA) and negative affect (NA) was measured using the Positive And Negative Affect Scale—mood State questionnaire (PANAS-S), to assess possible mood changes over the session. To track possible changes in mood valence and arousal during the session, participants were also asked to rate their current mood on an affect grid immediately after completing each task. Both of the mood measures were completed on a computer. The task order for every session can be seen in Figure 1.

### Auditory stimuli

Auditory stimulation was presented through in-ear headphones (Etymotic Research ER-4B microPro), which provide 35 dB external noise attenuation. All sound files (44 kHz, 16 bit) were digitally generated in Audacity and played from the test computer using E-Prime 2. Sound levels at output were calculated from the voltages delivered at the earphone input as measured with an oscilloscope (Type Tektronix TDS2002) and the earphone efficiency as provided by the earphone manufacturer (180 dB sound pressure level (SPL) for 1 Vrms in a Zwislocki coupler, ER-4 datasheet, Etymotic Research, 1992). As beats are best perceived with a carrier frequency between 300 and 600 Hz (Licklider et al., 1950; Oster, 1973), both binaural beat conditions (10 Hz and 40 Hz) were based upon a 340 Hz carrier frequency. This 340 Hz carrier tone was presented to both ears in the control condition. The alpha frequency (10 Hz) beat was generated by presenting a tone of 335 Hz to the left ear and a tone of 345 Hz to the right ear, while the gamma frequency (40 Hz) beat was generated by presenting a tone of 320 Hz to the left ear and a tone of 360 Hz to the right ear. In all conditions, white noise (20 Hz–10 kHz band filtered) was added to the signal in both ears to enhance the clarity of the beats (Oster, 1973).

### Eye blink rate (EBR)

Participants’ spontaneous EBRs were measured for 5 min at the start of each session using a BioSemi ActiveTwo system (BioSemi Inc., Amsterdam). During measurement of the blinks participants were not presented with any auditory stimuli. Spontaneous EBR was measured using six Ag/AgCL electrodes: two placed next to the outer canthus of each eye (measuring saccades), and two placed above and below the right eye (measuring the blink). Two electrodes placed on the mastoids served as a linked online reference. Participants were instructed to relax and look (but not stare) straight ahead at a paper with a fixation cross that was taped on the computer monitor. This monitor was turned off during EBR measurement. As EBR is stable during the day but goes up in the evening (after 8.30 pm; Barbato et al., 2000), participants were never tested after 7 pm. Blinks were identified automatically, and then manually checked for errors (such as noise segments wrongly identified as blink) in BrainVision Analyzer. Individual EBR was calculated by dividing the total amount of blinks during the 5 min measurement period by 5.

### Divergent thinking: Alternate Uses Task (AUT)

In this task, participants were to name as many uses for certain common household objects as possible. This task was scored on four components: originality, fluency, flexibility, and elaboration. However, as flexibility is most strongly and reliably connected to EBR scores (Akbari Chermahini and Hommel, 2010), we focused on this score, which reflects the number of different categories a participant uses in his or her answer for each item. For example, folding a hat of a paper or using it for origami counts as one category (folding), whereas writing a note on it counts as another (writing). We used a Dutch version of this task, which consisted of six items: *brick*, *shoe*, *paper*, *pen*, *bottle*, and *towel* (*baksteen*, *schoen*, *krant*, *pen*, *fles*, and *handdoek*, respectively). Per session, participants were given two items to solve in 10 min.

### Convergent thinking: Remote Associations Task (RAT)

In this task, participants were presented with three seemingly unrelated words (e.g., “market”, “star” and “hero”) for which they had to find a single compound word that could be associated with all three of these words (in this case “super”; creating the words “supermarket”, “superstar” and “superhero”). We used the Dutch version of this task, which consists of a total of 30 items (Cronbach’s alpha = .85; Akbari Chermahini et al., 2012). As our experiment consisted of three sessions per participant, we divided this task into three versions of 10 items each (Cronbach’s alphas = .70, .67, and .70), matched by the items’ discrimination value as reported in Akbari Chermahini et al. (2012). Participants were given 4 min to complete the 10 items.

### Positive and Negative affect schedule mood state questionnaire (PANAS-S)

This self-report mood scale consists of 20 items that provide a general measure of current mood in terms of PA and NA. Participants were given 10 positive (for instance, “interested” or “alert”) and 10 negative (for instance, “upset” or “guilty”) words, and had to indicate how applicable a word was to their current mood on a Likert scale between 1 (very little or not at all) and 5 (very or extremely). The PANAS-S was completed on a computer, where participants used the mouse to select an option on the Likert scale.

### Affect grid

Participants indicated their current pleasure and arousal level by means of a computer mouse, which served to place a single cross in an arousal × pleasure affect grid (Russell et al., 1989) presented on a computer monitor. The 9 × 9 grid was composed of a horizontal axis to code the current pleasure level (ranging from 1 [extremely unpleasant] on the left to 9 [extremely pleasant] on the right) and a vertical axis to code the current arousal level (ranging from 1 [low arousal] at the bottom to 9 [high arousal] at the top).

## Results

As a repeated measures analysis of variance (ANOVA) found no differences in EBR between the three sessions, *F*(2, 46) = 1.77, *p* = 0.18, we took the average across all three measures as an estimate of the individual EBR. To test whether binaural beats affected performance in the creativity tasks, repeated measures ANOVAs with auditory stimulation (Alpha, Gamma, Control) as within-participant factor were conducted.

**Figure 2:**
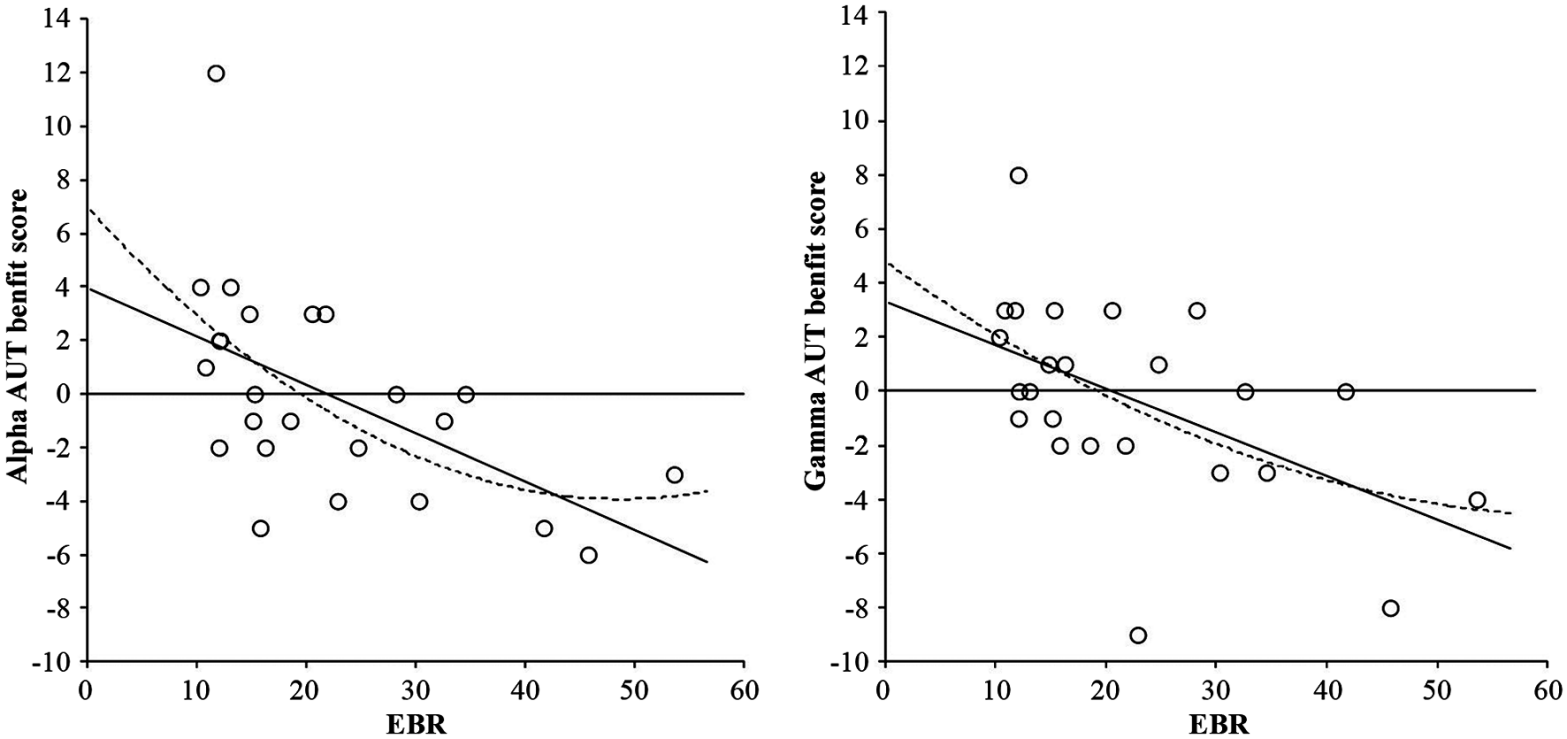
**Linear (solid line) and quadratic (dotted line) relationships between AUT flexibility benefit score from alpha frequency binaural beats and EBR (left-hand graph), and AUT flexibility benefit score from gamma frequency binaural beats and EBR (right-hand graph).** Benefit scores were calculated by subtracting performance in the control condition from performance in the binaural beat condition (either alpha or gamma).

The basic analysis of the AUT flexibility score (divergent thinking) showed no reliable effect, *F*(2, 46) < 1. However, adding the individual EBR as centered covariate (cf., van Breukelen and van Dijk, 2007) rendered the effect highly reliable, *F*(2, 44) = 5.22, *p* = 0.009, suggesting that the effect might be mediated by EBR. This was confirmed by regression analyses relating the individual Alpha benefit (performance in the Alpha condition minus performance in the Control condition) and the individual Gamma benefit (performance in the Gamma condition minus performance in the Control condition) to individual EBR. As shown in Figure 2, the relationships between both the Alpha benefit and EBR, *F*(1, 22) = 9.71, *p* = 0.005, and the Gamma benefit and EBR, *F*(1, 22) = 8.51, *p* = 0.008, followed highly reliable negative linear trends, while the quadratic trends explain lesser variance: *F*(2, 21) = 5.31, *p* = 0.014, and *F*(2, 21) = 4.23, *p* = 0.029, respectively. Interestingly, the distribution clearly crosses the zero line, suggesting that people with low EBRs (under 20 blinks per min) mostly benefit from both alpha, and benefit or are not impaired by gamma binaural beats, while people with higher EBRs do not benefit or are even impaired by binaural beat stimulation.

**Figure 3:**
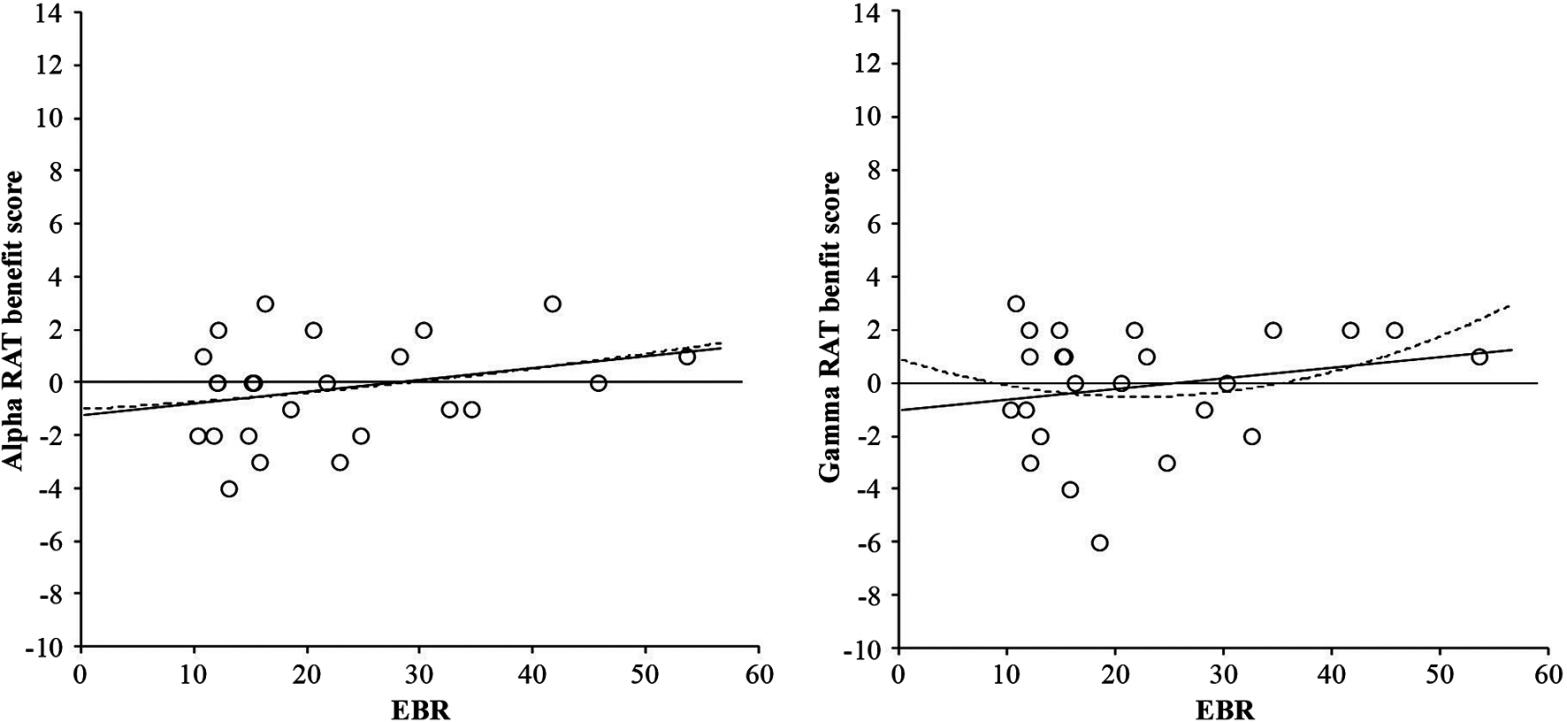
**Linear (solid line) and quadratic (dotted line) relationships between RAT benefit score from alpha frequency binaural beats and EBR (left-hand graph), and RAT benefit score from gamma frequency binaural beats and EBR (right-hand graph)**. Benefit scores were calculated by subtracting performance in the control condition from performance in the binaural beat condition (either alpha or gamma).

For the RAT score (convergent thinking) neither the basic ANOVA nor the ANCOVA with EBR as covariate yielded any reliable effect, *F*(2, 46) < 1. These non-significant relationships can be seen in Figure 3.

Given the assumption of a link between dopamine and mood or affect, we also explored whether the changes in performance were accompanied by changes in mood. This did not seem to be the case, however. For one, an (2 × 3) ANOVA of the NA score of the PANAS-S before and after the three sessions did not reveal any reliable effect, *p*s > .3. The same ANOVA of the PA PANAS-S scores produced a significant effect of time point, *F*(1, 23) = 11.07, *p* = 0.003 (indicating a slight reduction of positive mood from 29.9 to 28.3), but this effect did not interact with auditory condition, *p* > .18. The analysis of the affect grid data also found no indication for condition-specific effects on average pleasure, *F*(2, 46) < 1, or arousal, *F*(2, 46) < 1 scores.

## Discussion

The aim of this study was to investigate whether binaural beats, a form of cognitive entrainment, affect people’s creative performance, and whether such impact might be mediated by the individual striatal dopamine level, as assessed by means of EBR. The outcome provides a straightforward picture.

First, we found no evidence for any influence of binaural beats on convergent thinking, while divergent thinking was systematically affected depending on base-line EBR. This supports the assumption that convergent thinking, and other kinds of highly constrained top-down search processes, rely more on the frontal part of the frontal-striatal interaction constituting cognitive control (in the sense of Cools and d’Esposito, 2009), while divergent thinking, and other forms of mental flexibility, lean more towards the striatal part (Akbari Chermahini and Hommel, 2010; Hommel, 2012). Moreover, the observation of a differential effect on one of the two kinds of creative performance reinforces claims that human creativity is not a unitary function but consists of multiple components (Wallas, 1926; Guilford, 1967; Nijstad et al., 2010).

Second, we could not find any difference between the Alpha and the Gamma condition—both had the same kind and the same degree of impact on divergent thinking. This suggests that binaural beats do not so much trigger or facilitate a particular neural synchronization processes but rather support neuronal phase locking in general (Kuwada et al., 1979). For instance, they might impose some temporal structure on neural processes and thereby reduce cortical noise (Karino et al., 2006), which again may make task-specific processes that rely on neural communication and/or synchronization more reliable. In which frequency this temporal structure is operating might be less relevant.

Third, our findings clearly suggest that binaural beats do not represent a suitable all-round tool for cognitive enhancement. While participants with lower EBRs (20 blinks per min or lower) showed clear beat-induced benefits in divergent thinking, binaural beats impaired the performance of individuals with higher EBRs (20 blinks per min or higher; see Figure 2). As suspected, this suggests that beat-induced cognitive enhancement depends on the individual striatal dopamine level—an observation that parallels Akbari Chermahini and Hommel’s (2012) finding of equally selective mood effects on divergent thinking.

There are at least two possible, not mutually exclusive explanations for this observation. First, there is evidence that lower-than-average EBR levels are associated with less effective performance in divergent-thinking tasks, especially regarding flexibility (Akbari Chermahini and Hommel, 2010). Even though this difference just missed the significance criterion in our study (in the control condition, the flexibility scores of the low and high EBR groups were 10.58 and 12.83, respectively, *p* = .08), individuals with rather low striatal dopamine levels might have more room for improvement and are, thus, more sensitive to cognitive-enhancement procedures. For instance, it might be that binaural beats induce, or increase the size of phasic dopamine bursts, which might have a stronger impact in individuals with a relatively low tonic dopamine level. Individuals with a more suitable dopamine level may not need these extra or extra-sized bursts and may end up with more than optimal cortical noise. This would also suggest that EBRs mainly reflect tonic dopamine activity in the striatum, but this lies outside the scope of the current study and, thus, remains speculation for now.

Second, it might be that binaural beats do not operate directly on the individual dopamine level, be it tonic or phasic. Note that we did not find any systematic, beat-induced mood effects. To the degree that changes in dopamine levels are accompanied by changes in mood (Akbari Chermahini and Hommel, 2012), this might suggest that binaural beats facilitated or enabled processes that compensate for the individual lack of striatal dopamine. For instance, it might be that dopamine is functional in driving neural synchronization (Schnitzler and Gross, 2005). If so, a relatively low level of striatal dopamine may thus make it more difficult to set up synchronized neural states, and this difficulty may somehow be overcome through other, compensatory processes that are induced or facilitated by binaural beats. As speculated earlier, binaural beats may increase the temporal structure of idling neural activities and thereby reduce cortical noise, which again might facilitate setting up synchronized states. Again, it is conceivable that individuals with more optimal dopamine levels do not need, or may even be impaired by this alternative way to create the necessary synchronized states.

Irrespective of which of these two scenarios will turn out to be more realistic, it is clear that binaural beats do not represent a one-size-fits-all enhancement technique. They can be effective in enhancing brainstorm-like creative thinking in individuals with low striatal dopamine levels, but they can at the same time impair performance in exactly the same kind of task in others. On the one hand, this calls for more care in the propagation of binaural beats as a cognitive-enhancement method and a better understanding of the underlying neural and cognitive mechanisms. On the other hand, however, it also implies that previous failures to find positive effects of binaural beats on cognitive performance need not be taken as evidence against the efficiency of the manipulation. In fact, careful selection of individuals involving a systematic evaluation of their cognitive control profiles is likely to yield evidence of cognitive enhancement, even under conditions that proved ineffective by previous research.

## Conflict of interest statement

The authors declare that the research was conducted in the absence of any commercial or financial relationships that could be construed as a potential conflict of interest.
